# Event Related Potentials Index Rapid Recalibration to Audiovisual Temporal Asynchrony

**DOI:** 10.3389/fnint.2017.00008

**Published:** 2017-03-22

**Authors:** David M. Simon, Jean-Paul Noel, Mark T. Wallace

**Affiliations:** ^1^Neuroscience Graduate Program, Vanderbilt Brain Institute, Vanderbilt University Medical Center, Vanderbilt UniversityNashville, TN, USA; ^2^Vanderbilt Brain Institute, Vanderbilt University Medical Center, Vanderbilt UniversityNashville, TN, USA; ^3^Vanderbilt Kennedy Center, Vanderbilt UniversityNashville, TN, USA; ^4^Department of Hearing and Speech Sciences, Vanderbilt School of Medicine, Vanderbilt UniversityNashville, TN, USA; ^5^Department of Psychology, Vanderbilt UniversityNashville, TN, USA; ^6^Department of Psychiatry, Vanderbilt UniversityNashville, TN, USA

**Keywords:** audio-visual, multisensory, temporal, recalibration, EEG, plasticity

## Abstract

Asynchronous arrival of multisensory information at the periphery is a ubiquitous property of signals in the natural environment due to differences in the propagation time of light and sound. Rapid adaptation to these asynchronies is crucial for the appropriate integration of these multisensory signals, which in turn is a fundamental neurobiological process in creating a coherent perceptual representation of our dynamic world. Indeed, multisensory temporal recalibration has been shown to occur at the single trial level, yet the mechanistic basis of this rapid adaptation is unknown. Here, we investigated the neural basis of rapid recalibration to audiovisual temporal asynchrony in human participants using a combination of psychophysics and electroencephalography (EEG). Consistent with previous reports, participant’s perception of audiovisual temporal synchrony on a given trial (t) was influenced by the temporal structure of stimuli on the previous trial (t−1). When examined physiologically, event related potentials (ERPs) were found to be modulated by the temporal structure of the previous trial, manifesting as late differences (>125 ms post second-stimulus onset) in central and parietal positivity on trials with large stimulus onset asynchronies (SOAs). These findings indicate that single trial adaptation to audiovisual temporal asynchrony is reflected in modulations of late evoked components that have previously been linked to stimulus evaluation and decision-making.

## Introduction

Events in the natural environment generate signals in different sensory modalities, and combining these signals through multisensory integration can confer substantial behavioral and perceptual benefits (Murray and Wallace, 2012). An example of such facilitation can be found, for instance, for speech signals, in which the presence of visual cues (i.e., lip movements) substantially improves speech intelligibility (Sumby and Pollack, 1954; Ross et al., 2007). Numerous investigations have indicated that these perceptual effects are strongest when sensory stimuli from multiple modalities occur within an interval of time surrounding true simultaneity; an epoch known as the temporal binding window (TBW; Slutsky and Recanzone, 2001; van Wassenhove et al., 2007; Wallace and Stevenson, 2014; Noel et al., 2016b).

However, the inherent differences in the propagation speeds of light and sound present a significant challenge for the nervous system in the binding of audiovisual signals that are generated by a single source; a challenge partly solved by the presence of a temporal window within which signals can be integrated and bound (Keetels and Vroomen, 2012). A further solution presumably employed by the central nervous system is to dynamically recalibrate these perceptual processes. Indeed, previous work has demonstrated such recalibration after prolonged exposure to a fixed temporal asynchrony (Fujisaki et al., 2004; Vroomen et al., 2004). This phenomenon of temporal recalibration manifests as a shift of the point of subjective simultaneity (PSS) in the direction of the presented asynchrony.

Mechanistically, temporal recalibration is conceived as resulting from changes in both early sensory processes as well as in later, putatively more cognitive or decisional processing stages. Specifically, repeated presentation of asynchronous stimuli results in attenuation of early evoked responses (Stekelenburg et al., 2011) and shifts in the phase of neural oscillations in sensory regions (Kösem et al., 2014). This attenuation of early evoked responses has been proposed to represent a reweighting of the respective sensory inputs (Correa et al., 2006). In addition to these changes in early processing stages, however, later components of electrophysiological signals, occurring approximately 450 ms after stimulus onset, have also been reported to exhibit reduced voltage after prolonged sensorimotor temporal adaptation (Stekelenburg et al., 2011). These late components have previously been tied to stimulus evaluation as well as evidence accumulation and decision-making (O’Connell et al., 2012; Twomey et al., 2015). The presence of both low-level (i.e., sensory) and high-level (i.e., decisional) effects during prolonged adaptation thus suggest that temporal recalibration occurs at multiple levels of the processing hierarchy.

Recently, temporal recalibration has been demonstrated to occur not only after prolonged adaptation to asynchronous audiovisual signals, as exposed above, but also on a trial-by-trial basis (Temporal dimension: Van der Burg et al., 2013; Spatial dimension: Shams et al., 2011; Wozny and Shams, 2011). Such rapid recalibration is presumably critical for adaptive function, as recalibration processes must operate on rapid time-scales in order to provide optimal behavioral and perceptual benefits in response to dynamically changing stimuli. The ecological value of rapid recalibration is further reinforced by the asymmetric nature of the process, in which asynchronous events induce greater recalibration when they have a temporal structure consisting of a leading visual stimulus (Van der Burg et al., 2013; Van der Burg and Goodbourn, 2015). That is, rapid recalibration on a given trial (t) is stronger when preceded by a visual-leading stimuli on the previous trial (t−1)—the natural temporal structure of events in the real world—than when preceded by an audio-leading presentation. Finally, emerging evidence suggests that this rapid recalibration process may be altered in individuals with multisensory temporal dysfunction, such as Autism Spectrum Disorder (ASD; Noel et al., 2017; Turi et al., 2016), in which there is a growing body of evidence for the presence of multisensory temporal dysfunction in general (Stevenson et al., 2014). Indeed, the differences in audiovisual temporal acuity in ASD may play an important and underappreciated role in building social communicative representations (Wallace and Stevenson, 2014).

As described above, although the neural correlates of longer-term recalibration are beginning to be elucidated, relatively nothing is known about the neural processes subserving *rapid* temporal recalibration effects. Thus, the present study was designed to examine this phenomenon using an audiovisual simultaneity judgment (SJ) task while concurrently measuring electroencephalography (EEG). Our results indicate that the magnitude of relatively late event related potentials (ERPs; >125 ms post second-stimulus onset) are affected by the temporal ordering of audiovisual stimuli on the previous trial. The spatiotemporal nature of these differences suggests that rapid recalibration to audiovisual asynchrony is reflected in higher order sensory and/or decisional processes, rather than initial and early sensory processing.

## Materials and Methods

### Participants

Thirty-two participants (16 women) right-handed participants with a mean age of 20.5 years (±2.89) participated in the study. All participants reported normal or corrected-to-normal vision and normal hearing. Six participants were excluded from analysis due to either excessive motion and blink artifacts resulting in >50% of trials being rejected (*n* = 4) or poor psychometric fits that precluded analysis (*n* = 2) leaving a total of 26 analyzed participants (13 women). This study was carried out in accordance with the recommendations of Vanderbilt University Behavioral Sciences Committee with written informed consent from all subjects. All subjects gave written informed consent in accordance with the Declaration of Helsinki. The protocol was approved by the Behavioral Sciences Committee.

### Psychophysical Task

**Figure 1:**
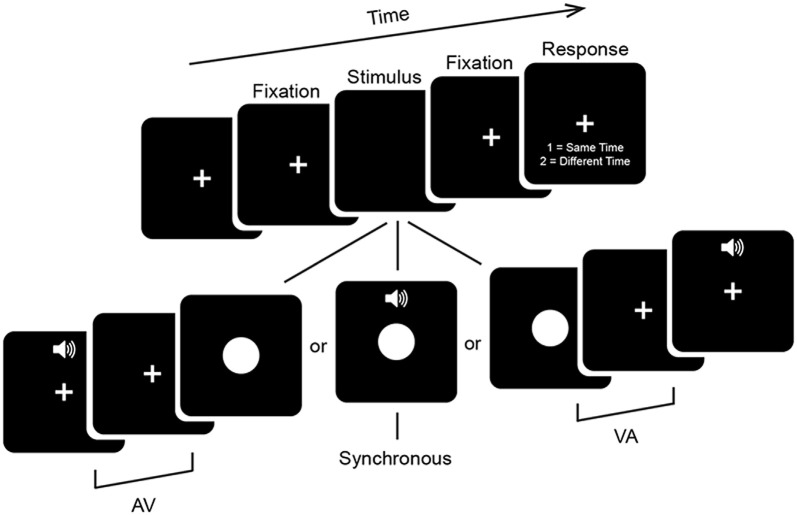
**Psychophysical task.** Trials began with a fixation period (see “Materials and Methods” Section for details). Audiovisual stimuli were then presented at one of seven stimulus onset asynchronies (SOAs) ranging from auditory leading by 300 ms (A300V) to visual leading by 300 ms (V300A) in increments of 100 ms. Trials were followed by a 650 ms fixation period and then a response screen which lasted until a response was given. The fixation cross was displayed continuously throughout the experiment.

Participants performed a 2 alternative forced choice SJ task (Figure 1). Visual (V) stimuli consisted of a white circle 11.4 × 11.4 cm (~6° of visual angle in diameter) presented centrally for 33 ms on a 24-inch monitor (ASUS VG248QE) with a refresh rate of 60 Hz. Auditory (A) stimuli consisted of a 33 ms 1000 Hz pure tone with 3 ms linear ramps (onset and offset) presented at approximately 65 dB from speakers on either side of the monitor. Stimuli were presented at a distance of 1 m via E-prime 2.0.10. Stimulus timing was confirmed with an oscilloscope to be both correct and to not depend on the stimulus ordering of the preceding trial. Trials began with presentation of a central fixation cross for between 650 ms and 1250 ms with a uniform distribution. This was followed by onset of an audiovisual stimulus with one of seven possible stimulus onset asynchronies (SOAs) ranging from A300V (A preceding V by 300 ms) to V300A (V preceding A by 300 ms) in increments of 100 ms. The second stimulus was followed by a 650 ms fixation period which was then followed by a response period which lasted until a response was given. Participants were instructed to use their right hand during the response period to indicate whether the stimuli were perceived to occur at the same time (i.e., simultaneously) or at different times (i.e., asynchronously) via keyboard button press. In other words, there was always a minimum period of 650 ms between the presentation of the second sensory stimulus and a motor response. This period minimizes motor contamination in the EEG signal, but also prevents meaningful analysis of reaction times. Participants were instructed to emphasize response accuracy over speed. Blocks consisted of 140 stimuli presented in pseudorandom order and participants completed a total of 12 or 13 blocks, for a total of 1680 or 1820 trials (240 or 260 per SOA). In addition, participants completed a single practice block of alternative SOAs ranging from A400V to V400A (in steps of 50 ms and excluding the SOAs administered in experimental blocks) before the main experimental blocks, which were not analyzed here. Total duration of the experiment including setup and breaks was under 2.5 h.

### Behavioral Data Analysis

We began our analysis by investigating whether the distributions for reports of synchrony as a function of SOA varied based on the ordering of the previous trial. We compiled for each participant synchrony report rates both independently from and depending on the temporal ordering (i.e., audio- or visual-leading presentation) of the preceding trial (trial t−1). Trials in which the t−1 stimulus was a synchronous presentation were excluded from this analysis. These distributions were then fitted with a single-term Gaussian psychometric function with free parameters of amplitude, mean and standard deviation (MATLAB fit.m), consistent with previous investigations of rapid audiovisual recalibration (Van der Burg et al., 2013; Noel et al., 2015). The mean of the best fitting distribution is taken as the PSS, while the standard deviation is taken as a measure of the TBW (Van der Burg et al., 2015; Noel et al., 2016a). In order to index the magnitude of single trial recalibration effects, the degree of change in PSS on the present trial was computed by subtracting the PSS for t−1 visual leads from that of t−1 audio leads (ΔPSS = PPS audio leading − PSS visual leading). Lastly, previous work has indicated that the overall magnitude of rapid recalibration effects (i.e., ΔPSS) correlates with the overall width of participant’s TBWs (Van der Burg et al., 2013; Noel et al., 2016a), and thus we correlated these values using linear regression (Pearson correlation).

Next, the approach described above was extended to individual SOAs by calculating reports of synchrony as a function of the specific SOA presented on trial t−1 (i.e., each SOA from −300 ms to +300 ms in steps of 100 ms). That is, reports of synchrony were not bifurcated based on whether the previous trial had been audio- or visual-leading, but instead were compiled according to the specific SOA on trial t−1. Thus there were a total of 49 “cells” (7 SOAs on the previous trial × 7 SOAs on the current trial), leading to a total of seven Gaussian fits per participant (corresponding with the seven possible SOAs on the previous trial). Changes across these fits index whether the magnitude of recalibration is dependent on the level of temporal lead on trial t−1.

### EEG Recording and Processing

Continuous EEG was recorded from 128 electrodes with a sampling rate of 1000 Hz (Net Amps 400 amplifier, Hydrocel GSN 128 EEG cap, EGI systems Inc.) and referenced to the vertex (Cz). Electrode impedances were maintained below 40 kΩ throughout the recording procedure. Data were acquired with NetStation 5.1.2 and further pre-processed using MATLAB and EEGLAB (Delorme and Makeig, 2004). Continuous EEG data were notch filtered at 60 Hz and bandpass filtered from 0.1 Hz to 40 Hz using a 4th order bi-directional zero-phase infinite impulse response (IIR) filter. Epochs from 300 ms before to 650 ms after onset of the first stimulus were extracted. That is, for the audiovisual stimuli with the greatest temporal disparity (i.e., 300 ms), the post-stimulus epoch was of 650 ms for the first stimuli and of 350 ms for the second. Artifact contaminated trials and bad channels were identified and removed through a combination of automated rejection of trials in which any channel exceeded ±100 μV and rigorous visual inspection. A mean of 1355 (79.3%), SEM = 42, trials were retained, while 2.65% (SD ± 1.67) of channels were removed per participant. Data were then recalculated to the average reference and bad channels were reconstructed using spherical spline interpolation (Perrin et al., 1987).

Subsequently, epochs were baseline corrected to the 200 ms pre (first) stimulus (i.e., −200 ms to 0 ms). ERPs were obtained by time domain averaging trials binned both as a function of SOA on trial t, and whether auditory or visual stimuli lead on trial t−1. We thus computed a total of 14 different ERPs (7 SOAs at trial t × 2 trial types at t−1). Trials in which the t−1 stimulus was a synchronous presentation were excluded from this analysis. There were no statistical differences in the number of trials retained for analysis across conditions (repeated measures ANOVA *f*_(6,25)_ = 0.47, *p* = 0.828) or lead types (repeated measures ANOVA *f*_(1,25)_ = 0.0004, *p* = 0.983). Statistical comparisons were made within each SOA between audio-leading and visual-leading using nonparametric cluster-based randomization testing as implemented in the fieldtrip toolbox (Oostenveld et al., 2011). This process was performed using data from 0 ms to 650 ms after onset of the first stimulus. Briefly, this process involves a first step in which ERPs for audio leading and visual leading conditions, at a specific SOA, are compared to one another, electrode-to-electrode and time point to time point via a paired sample *t*-test with *α* set to 0.05. Contiguous time points and electrodes yielding a significant result are then clustered in both space and time. Then, in a second step, the clusters found in the real data are tested against the cluster sizes found in random permutation of the conditions. Clusters in the real data larger than the 97.5th percentile (*a* = 0.025, equivalent to a two tailed test) of clusters in the permuted data are considered significant. This process corresponds to a 2-tailed significance test and controls for multiple comparisons in both space and time (Maris and Oostenveld, 2007). We set a minimum neighbor threshold of two significant electrodes for inclusion in the clusters and used a total of 10,000 permutations of the original data to assess statistical significance. To better illustrate the temporal dynamics of the effects we then selected the electrodes in each cluster when the cluster was at its largest size in terms of number of electrodes (A300V—509 ms, 23 electrodes; A200V—462 ms 26 electrodes; V300A—554 ms 24 electrodes) and averaged voltage over these electrodes at each time point from 0 ms to 650 ms after stimulus onset into cluster ERPs. Significance for cluster ERPs was again assessed using permutation testing. We used 10,000 permutations, *α* = 0.05 for cluster inclusion, and a more lenient *α* = 0.05 for permutation significance, corresponding with a 1-tailed significance test to correspond with our liberal selection of cluster electrodes (at any given time point many of the selected electrodes would not be individually significant, potentially leading to an underestimation of effects). Finally, we also extended this approach to the V200A condition using the same electrode cluster selected for V300A to determine if effects were qualitatively similar despite the lack of an identified significant cluster in the spatiotemporal analysis at this SOA (see “Results” Section).

## Results

### Trial-to-Trial Changes in Audiovisual Temporal Structure Recalibrates Temporal Perception

**Figure 2:**
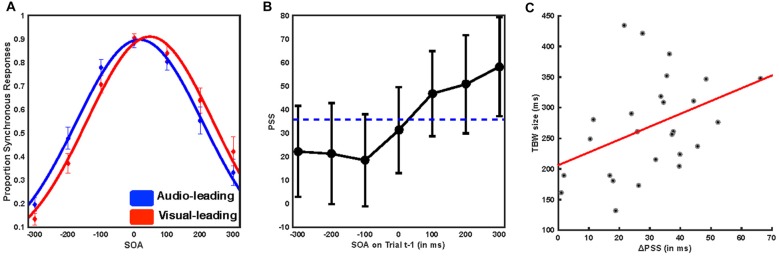
**Immediate temporal perception depends on the temporal structure of the preceding trial. (A)** Mean reported rate of synchrony at each of the seven SOAs binned by whether trial t−1 was audio leading (blue) or visual leading (red). Solid lines represent the Gaussian fit to the group average data. Error bars indicate the standard error of the mean. Trials preceded by a synchronous stimulus were excluded from this analysis. Note the shift in these distributions based on prior trial history. **(B)** Mean point of subjective simultaneity (PSS) as a function of SOA for the preceding trial. The horizontal dotted line indicates the mean PSS, while individual points indicate the PSS resulting from a Gaussian fit to all trials following the indicated SOA. Note that when audio-leads on the precedent trial, it is inconsequential by how much. On the other hand, the degree of recalibration monotonically increases as a function of the magnitude by which vision lead on the precedent trial. **(C)** Pearson correlation between temporal binding window (TBW) size and the shift in the PSS. Participants with larger TBWs showed greater rapid recalibration effects (i.e., changes in the PSS) *r* = 0.402, *p* = 0.042.

We first tested whether participants demonstrated rapid recalibration effects consistent with previous reports. Gaussian distributions were found to describe the reports of synchrony with a high level of fidelity (mean *R*^2^ of individual participant fits 0.979, SD ± 0.019, mean sum of squares error 0.0232 SD ± 0.0214, median log likelihood 469.788, SD ± 471.6). The amplitude (mean = 0.946, SEM = 0.025) of such distributions, a putative indicator of response bias, did not differ based on trial t−1 temporal ordering (Auditory leads, mean = 0.9371, SEM = 0.027; Visual leads, mean = 0.953, SEM = 0.026, paired sample *t* test, *t*_(25)_ = 1.8576, *p* = 0.075). We compared the PSS for all trials in which trial t−1 was auditory leading (Figure 2A, blue) with the PSS for all trials in which trial t−1 was visually leading (Figure 2A, red) using paired sample *t*-tests. We found that participants significantly shifted their PSS toward the lead type of the preceding trial (mean PSS 35.75 ms, SEM = 9.77 ms, mean ΔPSS = 30.26 ms, SEM = 2.97 ms, *t*_(25)_ = 9.984, *p* = 3.3 × 10^−10^; Figure 2A). We then extended this procedure to individual SOAs by fitting distributions according to the specific SOA on trial t−1. Goodness-of-fit again did not vary based on the nature of trial t−1 (all individual subject *R*^2^ > 0.828, repeated measures ANOVA, *f*_(1,25)_ = 1.93 *p* = 0.0791).

Further, the degree to which temporal recalibration occurred was highly dependent on both the type and magnitude of the temporal lead on trial t−1 (Figure 2B). When trial t−1 was visually leading, the PSS was shifted to both larger value (i.e., more visually leading) than the average (mean = 51.62 ms, SEM = 10.14 ms, paired sample *t*-test *t*_(25)_ = 9.008, *p* = 2.52 × 10^−9^), and the magnitude of the effect depended on the magnitude of the visual lead such that the greater the t−1 asynchrony the larger the recalibration effect (repeated measures ANOVA, *f*_(2,25)_ = 5.48, *p* = 0.007). On the other hand, when trial t−1 was auditory leading, the PSS was shifted to smaller values (i.e., less visually leading) than the average (mean audio leading PSS = 21.36 ms, SEM = 9.861 ms, paired sample *t*-test *t*_(25)_ = −10.468, *p* = 1.265 × 10^−10^), and the magnitude of the lead did not significantly impact the magnitude of recalibration (repeated measures ANOVA, *f*_(2,25)_ = 0.3, *p* = 0.741; see Van der Burg et al., 2013 for a similar effect). Lastly, we also found that there was a positive correlation between TBW size and recalibration magnitude, indicating that participants who displayed the largest TBWs also had the largest recalibration effects (*r*_(25)_ = 0.402, *p* = 0.042, Figure 2C). Our behavioral results thus replicate multiple aspects of previous psychophysical investigations of rapid temporal recalibration for audiovisual stimuli (Van der Burg et al., 2013; Noel et al., 2016a) and strongly indicate that the temporal structure of audiovisual stimuli on the preceding trial (i.e., t−1) impacts perceptual processing of the current trial (i.e., t).

### Audiovisual Temporal Structure on the Previous Trial Modulates Evoked Responses

**Figure 3:**
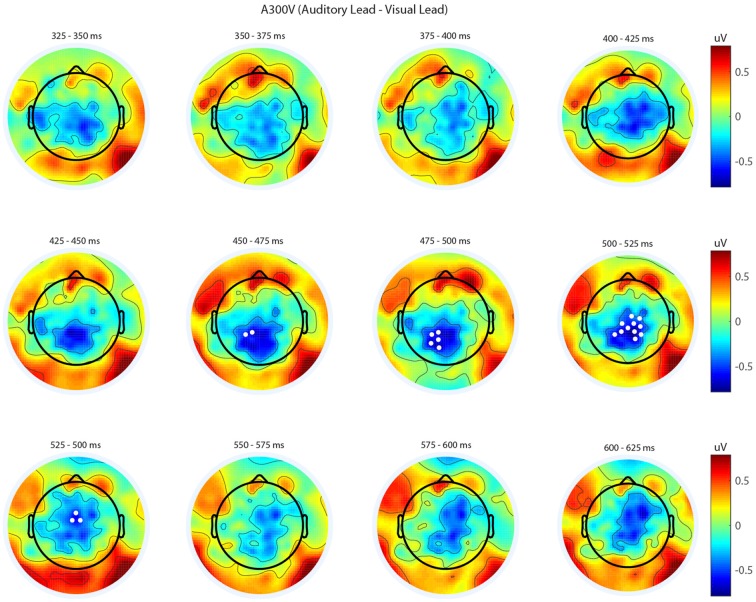
**Spatiotemporal representation of evoked differences in the A300V condition.** Cluster permutation testing indicated there was a single significant cluster (*p* = 0.0088). Voltages indicated are the difference between when trial t−1 was auditory leading and when trial t−1 was visual leading. Warm colors indicate voltage was greater when the preceding trial was an audio-leading trial. Cool colors indicate voltage was greater when vision-lead on the preceding trial. White circles indicate the locations of electrodes forming a significant cluster.

**Figure 4:**
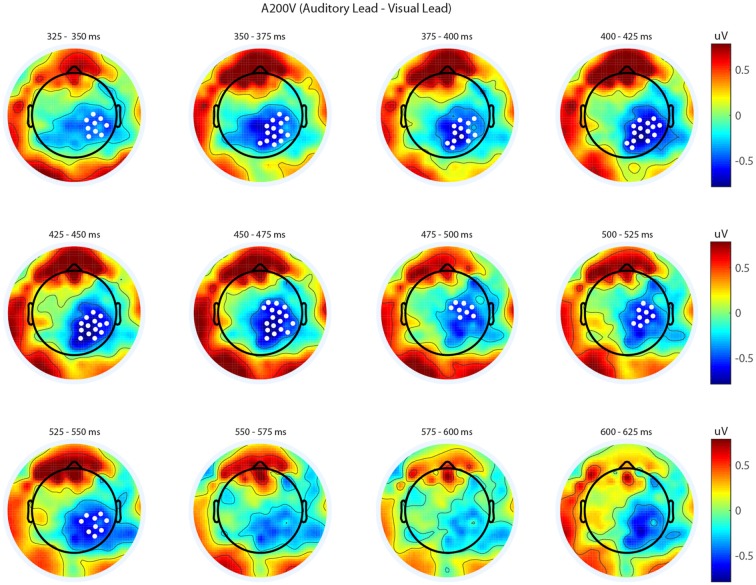
**Spatiotemporal representation of evoked differences in the A200V condition.** Cluster permutation testing indicated there was a single significant cluster (*p* = 0.0019). Voltages indicated are the difference between when trial t−1 was auditory leading and when trial t−1 was visual leading. Warm colors indicate voltage was greater when the preceding trial was an audio-leading trial. Cool colors indicate voltage was greater when vision-lead on the preceding trial. White circles indicate the locations of electrodes forming a significant cluster.

**Figure 5:**
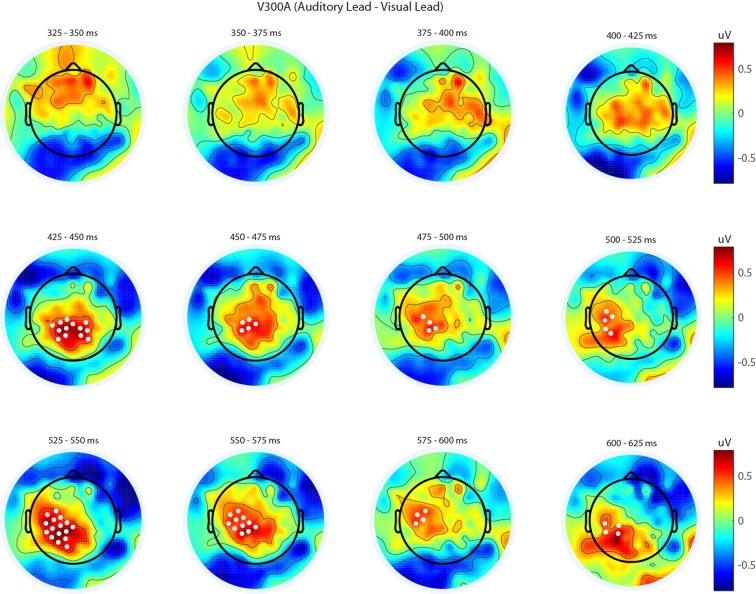
**Spatiotemporal representation of evoked differences in the V300A condition.** Cluster permutation testing indicated there was a single significant cluster (*p* = 0.00089). Voltages indicated are the difference between when trial t−1 was auditory leading and when trial t−1 was visual leading. Warm colors indicate voltage was greater when the preceding trial was an audio-leading trial. Cool colors indicate voltage was greater when vision-lead on the preceding trial. White circles indicate the locations of electrodes forming a significant cluster.

**Figure 6:**
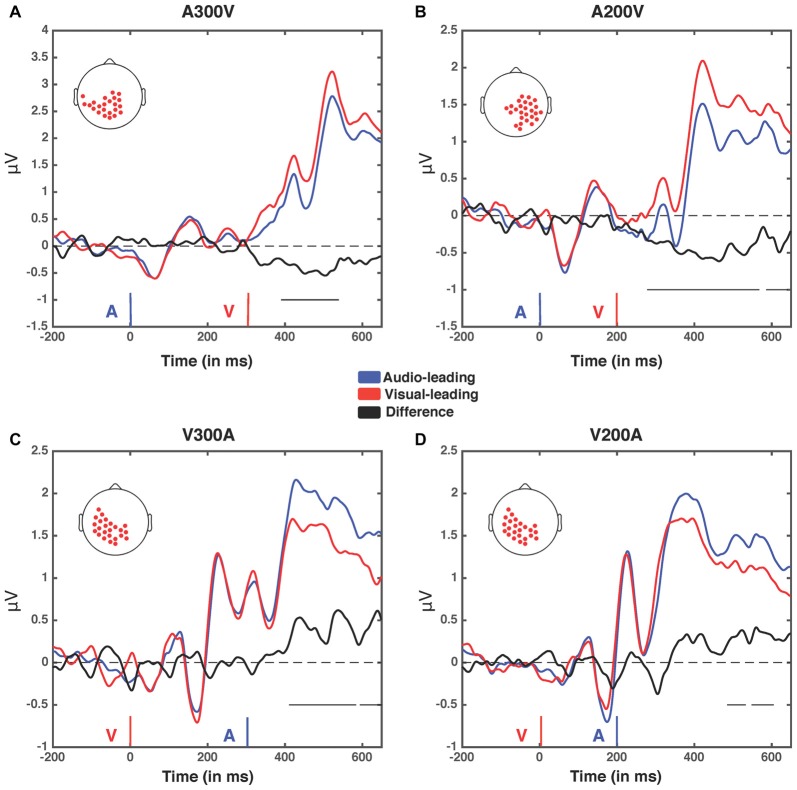
**Event related potentials (ERPs) for the four spatiotemporal clusters.** Electrodes contained within each cluster when it was at its largest spatial extent (in terms of number of electrodes) for the four greatest temporal disparities (A300V, A200V, V300A and V200A) were selected and voltage was averaged across them. The electrodes within the cluster in each condition are depicted by red dots on a scalp map (inset). Voltages when trial t−1 was an auditory lead are depicted in blue, while voltages when trial t−1 was a visual lead are depicted in red. The difference wave (audio-leading − visual-leading) is depicted in black. Onset of auditory (blue) and visual (red) stimuli are illustrated by vertical bars. Black horizontal bars at the bottom right of each panel indicate the temporal extent of significant clusters. **(A)** A300V—one significant cluster was found from 391 ms to 583 ms post-stimuli onset, *p* < 0.0001. **(B)** A200V—two significant clusters were found from 279 ms to 567 ms, *p* = 0.0002, and 587–640 ms, *p* = 0.0371. **(C)** V300A—two significant clusters were found from 412 ms to 583 ms, *p* = 0.0002, and 595–650 ms, *p* = 0.0147. **(D)** V200A—two significant clusters were found from 486 ms to 532 ms, *p* = 0.0394, and 549–604 ms, *p* = 0.0209. Note that electrodes averaged in this condition were selected from the V300A condition as the V200A condition did not produce a significant cluster.

**Figure 7:**
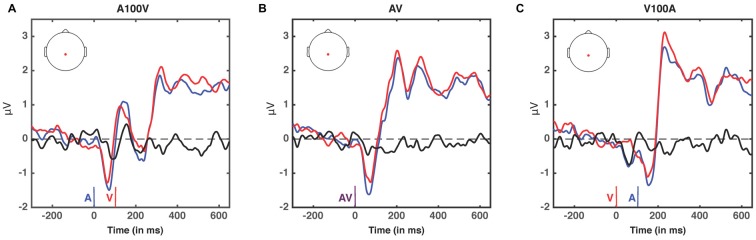
**ERPs at non-significant SOAs; A100V (A)**, AV **(B)** and V100A **(C)**. ERPs are presented for electrode 55, a centro-parietal electrode participating in each of the significant clusters (audio-leading vs. visual-leading at t−1, see Figure 6). ERPs when the preceding trial was audio-leading are depicted in blue, and ERPs when the preceding trial was visual-leading are depicted in red. The difference wave (audio-leading − visual-leading) is illustrated in black. Onset of auditory stimulus (blue), visual stimulus (red), and synchronous audiovisual stimulus (purple) are illustrated by vertical bars. No significant differences were found (all *p* > 0.17, randomization test).

We next analyzed evoked potentials to determine the neural correlates of the rapid recalibration effect. To do so, we utilized nonparametric permutation testing with cluster-based correction for multiple comparisons (Maris and Oostenveld, 2007). We performed testing over the entire post stimulus interval ranging from 0 ms to 650 ms for each SOA separately, contrasting when the previous trial was auditory leading and when the previous trial was visual leading. Clusters corresponding to significant differences between the two lead types were identified in a total of three of the seven SOA conditions (A300V, A200V and V300A), with a trend present in a fourth condition (V200A). These conditions all correspond to the outer (i.e., most temporally extreme) SOAs tested. In all three conditions which exhibited significant modulations in ERP as a function of the temporal structure of the preceding trial the identified clusters indicated that voltages at centro-parietal electrodes differed based on lead types. In the A300V condition a cluster was identified that demonstrated reduced voltages at centro-parietal electrodes when the previous trial was an auditory leading SOA as opposed to a visual leading SOA (cluster permutation test, *p* = 0.0088; Figure 3). In the A200V condition a similar cluster was identified indicating that again centro-parietal voltages were reduced when the previous trial was auditory leading (cluster permutation test, *p* = 0.0019; Figure 4). In contrast, in the visually leading V300A condition voltages were increased at centro-parietal electrodes when the previous trial was auditory leading (cluster permutation test, *p* = 0.00089), as compared to when the previous trial was a visual-leading stimuli (Figure 5). To better illustrate the temporal nature of differences in the ERPs, we selected the significant electrodes when each cluster was at its largest spatial extent (in number of electrodes) for each condition and averaged voltages across them. These cluster ERPs were then evaluated using cluster permutation testing over the entire post stimulus interval to evaluate for significant difference (Figure 6; for completeness we include in Figure 7 waveforms for the non-significant SOAs at electrode 55, a centro-parietal electrode participating in all significant clusters). In all conditions, these cluster ERPs were significant at late time points (A300V—391–538 ms, *p* < 0.0001; A200V—279–567 ms, *p* = 0.0002 and 587–640 ms *p* = 0.0371; V300A 412–583 ms, *p* = 0.0002 and 595–650 ms, *p* = 0.0147). Extended to the V200A condition the cluster defined in the V300A condition yielded qualitatively similar but weaker results (486–532 ms, *p* = 0.0394 and 549–604 ms, *p* = 0.0209).

Importantly, all three of the identified significant clusters had onset times for significant voltage differences which began approximately 125 ms after the second stimulus (e.g., the cluster for A200V appears about 100 ms earlier than the cluster for the A300V condition). This seemingly indicates that the timing of significant differences is temporally linked to the onset time of the second stimulus, rather than that of the first stimulus. Combined with the spatiotemporal distribution of the effects described above, appears to indicate that the reported physiological differences likely arise from decisional processes, rather than (early) sensory-linked processing differences. This inference is reinforced by the reversal of the direction of the effect corresponding with a reversal in stimulus order, in that voltages for auditory leads are decreased on auditory leading trials but increased for the visually leading trials. This relatively late timing is consistent with the concept that these late components reflect processes comparing stimulus structure across trials. Similarly, the order specific magnitude reflects that voltage differences seemingly index processes linked to stimulus evaluation, rather than activity linked directly to early sensory processing.

## Discussion

Previous work has indicated that recalibration to temporal asynchrony in audiovisual events occurs on both prolonged (Fujisaki et al., 2004; Vroomen et al., 2004) and rapid (Van der Burg et al., 2013) timescales, and that these processes are behaviorally dissociable (Van der Burg et al., 2015). Although prior work has begun to examine the neural underpinnings of prolonged audiovisual temporal recalibration, to the best of our knowledge, there is no report on the neural correlates of rapid (e.g., single trial timescale) temporal recalibration. Here we investigated the neural basis of rapid audiovisual temporal recalibration by examining the impact of the previous trial’s temporal structure on perceptual performance and electrophysiological responses. Behaviorally, we replicated multiple aspects of previous psychophysical investigations by showing that participant’s perceptual reports of synchrony were strongly influenced by the temporal structure of the immediately preceding stimuli (Van der Burg et al., 2013; Noel et al., 2016a, 2017). We add to the understanding of rapid recalibration by demonstrating, physiologically, that the temporal structure of stimuli on the previous trial influences evoked brain responses on the current trial. These effects were restricted to time points substantially after the onset of the second stimulus (>125 ms), appeared over centro-parietal electrodes, and were only evident for large temporal asynchronies.

### The Neural Signature of Rapid Recalibration Is Restricted to Late Evoked Components

The neurophysiological changes associated with rapid recalibration are seen quite late after the onset of the second stimulus. Modulation of late ERP components with a similar spatiotemporal pattern have previously been reported in prolonged sensory-motor temporal adaptation experiments (Stekelenburg et al., 2011). In this respect, we corroborate previous findings indicating that late processing stages are impacted by perceptual adaptation. Importantly, however, previous prolonged adaptation experiments also report attenuation of early responses (Stekelenburg et al., 2011), which is not present in our single trial adaptation results. These physiological differences can be readily explained by previous behavioral work demonstrating that rapid and more cumulative temporal recalibration function independently, suggesting that they should be dissociable at the neural level (Van der Burg et al., 2015). Our results seemingly indicate that this dissociation is present by linking the attenuation of relatively late amplitudes of ERPs, and seemingly not early responses, to rapid temporal recalibration.

It has previously been suggested that temporal recalibration may in fact represent multiple distinct stages of perceptual plasticity; a first step in which audiovisual temporal acuity (as captured in the concept of a TBW) enlarges (and thus in which the variability in the estimate of a PSS is enlarged) and a second stage in which the TBW contracts around a new PSS (Navarra et al., 2005). The process of establishing a new PSS, thus, would rely on the ability to first change the criterion for assessments of simultaneity (which would be reflected as a change in rapid recalibration and indexed by late electrophysiological changes) followed by a genuine recalibration of perceptual systems (which would arguably be indexed by earlier electrophysiological changes). This first stage likely represents a change in decisional processes, which have been linked to late centro-parietal ERP components consistent with the differences we observe (O’Connell et al., 2012; Kelly and O’Connell, 2013; Twomey et al., 2015). The second stage would then represent plasticity in sensory representations themselves, and manifest as changes in earlier evoked response components. Our results suggest that rapid recalibration may be most strongly linked to decisional processing stages, while prolonged recalibration is likely to necessitate changes at more sensory level of analysis. In the current effort we undertook no explicit temporal adaptation procedure and the nature of the transition from single trial effects manifested in relatively late components to cumulative effects visible in early components remains an exciting avenue for future research.

### Asymmetry in Neural Effects Mirrors Asymmetry in the Environment

A second main finding is that the neural effects we report demonstrate some asymmetry. The nature of the immediately preceding trial (in terms of audio-leading or visual-leading) had a significant impact on the processing of current audiovisual stimuli at auditory leads of 300 ms and 200 ms (A300V and A200V), with the largest impact at a lead of 200 ms. For visual leads, on the other hand the preceding trial only significantly impacted current processing at leads of V300A ms, with weak effects seen for V200A. No rapid recalibration effects were found for the small audiovisual asynchronies (<100 ms) used in the current experiment. Initially, these neurophysiological findings might appear to run counter to the behavioral findings (Figure 2C), where the magnitude effects were only seen for stimuli in which vision leads. That is, the degree to which PSS shifts is influenced by the magnitude of the lead on the preceding trial if it is a visual lead, but not if the preceding trial is an audio lead. Such a finding, however, makes strong ecological sense in that visual inputs leading auditory inputs is the common temporal structure for an audiovisual stimulus derived from a single location or event as a result of the difference in propagation times for light and sound. Thus, differences in the SOA generating the most significant ERP differences may lie in the strength of Bayesian priors (Stocker and Simoncelli, 2006); neural rapid recalibration effects were more readily observed for the audiovisual asynchronies less encountered in our daily lives.

An important observation is that asymmetry in the manner in which audio- and visual-leads are processed is a well-documented feature of multisensory integration, as previous reports have highlighted this feature in multisensory perceptual thresholds (Stevenson and Wallace, 2013) and in aspects of multisensory perceptual plasticity (Powers et al., 2009; Van der Burg et al., 2013). However, all previous reports indicate that visually leading trials drive plasticity the most strongly, are the most variable across participants, and are also the most amenable to perceptual plasticity on both short and long time scales. That is, to the best of our knowledge, the current finding represents the first to describe a multisensory plasticity effect that is more readily observed under circumstances when auditory signals lead visual signals. As noted above, this makes good ecological sense as rapid recalibration can be conceived of as a measure of (trial-by-trial) variability, and the natural statistics of the environment would dictate sparser sampling of auditory leading circumstances. EEG responses to audio-leads might thus be more variable than EEG responses to visually leading inputs. Indeed, recent theoretical accounts have cast the process of temporal recalibration under the light of Bayesian inference, a view that is in line with the observation that ERPs for audio-lead trials are more strongly impacted by the nature of the preceding trial than visual-lead ERPs are (Stocker and Simoncelli, 2006).

### Rapid Recalibration as a Manifestation of Changes in Decisional Processes

An intriguing and attractive explanation for the effects we observe is that when the past stimulus and the current stimulus have a similar temporal structure the boundary for a perceptual decision (Gold and Shadlen, 2007) is reached more easily. This reduction in required information to support a decision would then result in attenuation of late components that have been linked to evidence accumulation (O’Connell et al., 2012). The timing of the differences we observe is also consistent with recent reports indicating that decisional information regarding sensory timing appears earlier than was previously thought (Baumgarten et al., 2016). The decisional nature of this asymmetric modulation and its behavioral consequences is reinforced by the specificity of the rapid recalibration effect across the different SOAs. Significant rapid recalibration effects were present only when the current trial was presented with a relatively large temporal offset, and not for synchronous or moderately asynchronous (A100V and V100A) trials. As these late centro-parietal components are believed to be related to decisional processes (O’Connell et al., 2012), differences in these components may be enhanced for stimuli that are processed less automatically. Indeed, previous fMRI work has demonstrated that processing effort is enhanced by increasing levels of asynchrony (Stevenson et al., 2010). Consequently, increased effort and reduced automaticity may result in the recruitment of more neural resources to support a decision in regard to stimulus “binding” and thus increased signals within the relevant ERP components.

A closely related interpretation of the EEG results is that the attenuation/enhancement of ERP amplitudes we report serves as a marker of synchrony/asynchrony decisions on the current trial. In this framework, voltage differences would be attributable to the decisional outcome. The current experiment cannot rule out this possibility, as any split in terms of perception or lead type leads to similarly imbalanced “binning” of the other category due to the link demonstrated in our behavioral results between lead-type on the previous trial and decisional outcome on the current trial. The reported ERP results might thus reflect a combination of the actual decision (on trial t) and perturbations of decisional process (based on trial t−1). While we are unable to disentangle these explanations in our design, this yields exciting hypotheses for future work aiming to disentangle perceptual decision making from perceptual outcomes, and whether the effects we observe are cumulative (i.e., whether two trials in a row of a specific lead further magnifies the effects).

### Caveats and Future Directions

Although we found differences associated with rapid recalibration solely in relatively late components of ERPs, this does not rule out putative effects in early processes. Evoked responses are restricted to examining time locked neural responses (Makeig et al., 2004), and do not capture changes in induced brain activity or connectivity. Importantly, previous work indicates that relative phase alignment forms a distributed code for temporal relationships during prolonged audiovisual recalibration (Kösem et al., 2014). Similarly, these indices of oscillatory brain activity appear to be particularly dysfunctional in ASD (Simon and Wallace, 2016), and individuals with this disorder demonstrate both impaired multisensory temporal acuity (Stevenson et al., 2014) and altered audiovisual rapid recalibration (Noel et al., 2017; Turi et al., 2016). Due to the limited spatial resolution of EEG we are unable to determine in this design if changes in the relative phase of cortical oscillations occurs during rapid recalibration. Similarly, we are unable to completely rule out the possibility that changes in early sensory ERP components might make some contribution to the effects we find. Indeed, components 125 ms post-stimulus onset could putatively reflect both sensory and decisional processes.

Other network properties such as spike correlation are similarly unresolvable at the spatial scale of EEG (Nunez and Srinivasan, 2006) and have been hypothesized to reference perceptual decisions against past events (Carnevale et al., 2012). Additionally, the lateralization of effects cannot be strongly interpreted as potentially lateralized responses attributable to the sensory stimulus or motor preparation may be intertwined with the modulated responses. Future studies employing methods capable of examining cortical phase and precise spatial localization, as well as animal model work designed to probe and manipulate activity within the responsible networks, are needed in order to better elucidate the degree to which these effects contribute to rapid temporal adaptation. Additionally, we note that recalibration effects in early sensory ERP components may simply be too small for the power in our study to detect, as single trial adaptation would be expected to be of a smaller magnitude than prolonged adaptation. Future work designed to capture the temporal dynamics of decision-making using measures such as reaction time and participant confidence ratings may also further elucidate these effects in both typical and atypical development.

In summary, we provide the first evidence that single trial audiovisual temporal recalibration modifies late evoked neurophysiological components (i.e., >325 ms post onset of the first stimulus and >125 ms post onset of the second stimulus) that have been previously linked to decision-making processes. Furthermore, the observed neural effects were somewhat asymmetric, further corroborating the ecological validity of rapid temporal recalibration. Interestingly, from a behavioral perspective, the magnitude of the asynchrony on the previous trial drives perceptual recalibration more readily when vision leads audition. At a neurophysiological level, an inverted asymmetry is observed—audio-leads are more strongly impacted by the nature of the preceding trial than visual-leads are. Together, this complementary behavioral-electrophysiological asymmetry is interpreted to mean that the perceptual representations of visually-leading stimuli are more stable (i.e., less variable or amenable to change), and thus represent a stronger driving force for temporal recalibration at the level of the single trial.

## Author Contributions

DMS and MTW designed the experiment. DMS and J-PN collected and analyzed the data. DMS, J-PN and MTW wrote and revised the manuscript; approved the final manuscript.

## Conflict of Interest Statement

The authors declare that the research was conducted in the absence of any commercial or financial relationships that could be construed as a potential conflict of interest.
